# Anopheles and Malaria

**Published:** 1904-06

**Authors:** A. Laveran


### ANOPHELES AND MALARIA.*

BY A. LAVERAN.

Translated from the French by Louis M. Warfield, M.D., Savannah, Ga. (Formerly Medical House Officer, the Johns Hopkins Hospital).

It is demonstrated to-day that the hematozoon of malaria accomplishes one part of its evolution in the body of the anopheles and that those of the culicides which have become infected, after having sucked malarial blood, are capable of propagating malaria. Investigators have followed, day by day, the transformations of the hematozoon in the anopheles, and they have given typical malarial fever to a certain number of healthy individuals, by causing them to be bitten by artificially infected anopheles; one can now affirm that the demonstration leaves nothing to be desired.

From the fact that the anopheles are capable of inoculating malaria, one can not conclude that this is the only means of propagation of the illness. Many diseases have multiple modes of infection; malaria itself can be inoculated *artificially* from a sick to a well man without the mediation of the anopheles. Some have objected to the doctrine of the propagation of malaria by the anopheles, or, to state it briefly, "doctrine anophelienne," that, in certain unhealthy localities there were no culicides, and that, in healthy places, one found, on the contrary, culicides in large numbers, and notably the anopheles.

A vast research was necessary in order to establish the relations existing between the culicides and malaria and to judge of the value of the objections made against the anophilism doctrine. This reseach, which has been undertaken in many lands, is still being pursued; but the results which it has given are already important enough to deserve a resume and a coordination.

In a general way, the results of the inquiry have been quite favorable to the anophilism doctrine. For my part, I can affirm that all my numerous observations made since 1899 have added support to this doctrine. I have recently published the resume of my re-

*Read before the Georgia Medical Society, April 27, 1904.

searches, and I shall content myself here with stating my conclusions only:

"I have proved the existence of anopheles in all the malarious lands that I have visited and in all the samples of mosquitoes sent to me which were captured in malarial places; the number of anopheles in a given locality is found to be almost always in direct ratio to the frequency of the fevers."

In the warm countries, as in the temperate lands in the North, as well as in the middle of Europe, one can affirm that it is the rule to find anopheles in all the malarious localities.

The anophilism doctrine is quite in accord with the epidemiology of the malarial fevers.

In the temperate countries, where the seasons are well defined, the first attacks of malarial fevers show themselves at the beginning of summer, precisely at the time when the anopheles come out from their winter hibernation and begin to multiply. It is about the last of June that one finds in Algiers and Italy the first infected anopheles and it is precisely at this time that the first cases of fever develop; the number of infected anopheles increases in July and August and then decreases. There is, as numerous observers have noted, a remarkable relation between the evolution of the anopheles and that of the fevers.

In the tropics, it is during the rainy season that one finds the greatest number of anopheles.

The inquiry has also revealed the fact that the number of species of anopheles is far greater than was thought to be the case at first. Theobald in his monograph on the culicides, published in 1901, describes forty-five species of anopheles and, since then, quite a number of new species have been discovered. This multiplicity of species complicates the question, because all the kinds of anopheles do not seem to be able to carry the infection. I shall return later to this point.

In Europe, it is *A. maculipennis* Mergin, which is the chief carrier of infection; next comes *A. bifurcatus* Lin., *A. superpictus* and *A. pseudopictus* Grassi. These four species are concerned in the propagation of malaria.

In order that the anopheles should be capable of giving malaria, it is necessary that they themselves should be infected by biting individuals ill with malaria.

The anopheles are not infected when born in the most unhealthy marshes, even though they are hatched from eggs laid by infected females. Besides, malaria being a disease peculiar to man, the anopheles can not become infected by biting animals. It does not suffice then, that the anopheles should be in a locality for malaria to be propagated; it is necessary that the mosquitoes should infect themselves by sucking blood containing the malarial parasite. From this point of view, the recent inquiry into the conditions of development of endemic malaria has given some very interesting results.

We have known for some time that, in malarious countries, a very large proportion of the children are infected.

At Borneo, in the unhealthy villages, Néuwenhius has found splenic tumors in 80 per cent. of the children examined.

The new and important fact which the labors of R. Koch have made known is that, in native children, in the malarial countries, one very frequently finds the hematozoa in the blood.

In a village situated near a morass in Batavia, among one hundred and eighty-nine children examined, forty-five had malarial parasites in the blood, 22.8 per cent.; in children under one year, the proportion was raised to 41 per cent.

In a healthy locality near Tosari (5,400 feet altitude) among eighty-two children examined, not one was infected; cases of malaria at Tosari were found, but only in those who had gone down to malarious localities. As Koch says, it is in examining the children that one can arrive at the degree of malarial infection of a community.

The native children among whom the hematozoon of malaria is found have few acute manifestations; the infection assumes among them a chronic form very favorable to the infection of the anopheles on account of the persistence of the parasites in the blood; the absence of treatment too assists this infection.

It could be held according to the statements of Koch that the malaria is *latent* in the native children; it is not, however, so. When one examines the spleen, it is found increased in size (a fact long recognized); fever is not rare, but among the children it is not of the regular types and not so easy to diagnose as the types of fevers of the ordinary kind in the adult.

Koch seems to have considerably exaggerated the immunity which the attack of malaria confers in childhood, but this question does not enter into the scope of this article.

In German New Guinea, as in Batavia, Koch has frequently found the parasite in children under five years who live in malarious districts.

Stephens and Christophers, on the Gold Coast and in India have verified the common occurrence of the hematozoa in the native children. In a village near Accra, among forty-one children examined, twenty-five were infected; in one house among seven children four were infected. In another village among sixteen children examined, eleven were infected, of whom eight had crescent forms. In a military encampment, among twenty-five children examined at random, seventeen were infected, of whom six had crescent bodies.

The infection is moreover commoner among the younger children; in the first village mentioned above the infection was shown to be present in ninety per cent. of children under two years, in fifty-seven per cent. from two to eight years, in twenty-eight per cent. from eight to twelve years; above twelve years the infection was rare.

At Calcutta, Stephens and Christophers have not found infected children, while at the foot of the Himalaya, in certain very unhealthy villages, the number of infected children reached forty to seventy-two per cent.

Likewise in India, James has shown that, in certain unhealthy localities seventy-five per cent. of the children under ten years had malarial parasites in the blood. The infected children have, James asserts, hypertrophied spleens, and they often present an evening rise of temperature.

Zieman has shown the frequency of malaria among the negro children of the Cameroons (West Africa). Children from one to five years were infected in the proportion of thirty-seven per cent.; from five to ten years, eighteen per cent.; from ten to sixteen years in the proportion of twenty-one per hundred.

Steuber has made the same statements in German East Africa. The negro children, he says, almost all suffer from fever. The spleen is generally increased in size. At Tabria the hypersplenitis has been found in seventy-five per cent. of the negro children.

Zieman and Steuber affirm that this attack of malaria in the young confers no immunity. At Daressalam, Steuber has often found malarial parasites in the blood of adult negroes.

. . . . . . . .

Some observers urge against the doctrine of anophilism the fact that mosquitoes are absent in certain unhealthy localities. This objection would be serious if the actuality of the alleged facts was demonstrated; but every time that these facts have been the objects of serious enquiry, it has been shown that in the localities designated culicides and anopheles in particular, have been found, or that the cases of malaria observed had been contracted in other localities.

Such has been the result of the research undertaken at Batavia by Koch, a research quite favorable to the doctrine of anophilism.

Two localities of Corsica, Lumio, near Calvi, and Ponte-Leccia, have been designated as unhealthy, in spite of the complete absence of mosquitoes. In the month of September, 1902, I visited these localities in company with Dr. Batteste, and in broad day, we captured *anopheles maculipennis* in several dwellings; we showed in Lumio and in the valley of Casa-Luna (designated too as unhealthy, although free from mosquitoes) the existence of pools containing numerous culicides larvæ.

Dr. Soulié has cited among the swampy areas in Algeria free from anopheles the villages of Marengo and Montebello. Dr. Sergent visited in May last these places. At Marengo, he found in two pools of water, thirteen hundred yards and nine hundred yards from the first houses of the village, numerous larvæ of *A. algenensis*. At Montebello he found at three different points to the southwest of the village, in a little canal which emptied into the irrigation canal from lake Haloula, numerous larvæ of *a. maculipennis*.

It is often difficult to find larvæ of anopheles and it is not necessary that the mosquitoes should be numerous in a district in order to propagate malaria. Let us add that the infected anopheles can be transported in various ways from one locality to another.

In Italy the transportation of anopheles by means of public carriages has often been shown. According to Vivarate, there are not in Venice stagnant waters suitable for the development of anopheles. These mosquitoes would be imported by the boats carrying market produce, fodder, straw and especially marsh-reeds.

The experiments made to infect the culex raised from larvæ in captivity by making them bite patients ill with malaria have so far failed.

Dr. Montor, of Francesco, has advanced the view that the culex, themselves, without becoming infected could transport the parasite of malaria and inoculate as one would do with a lancet. In support of this hypothesis, the author has cited no fact proving it.

Another objection to the doctrine of anophilism has been deduced from the presence of anopheles, at times numerous in healthy localities, or those localities formerly unhealthy, have been drained without the coincident disappearance of the anopheles. The researches of Nuttall L. Cobbet and Strangeways Pigg in England show that anopheles are found in all districts formerly malarious, and also in localities where endemic malaria has never been found, three species of anopheles have been encountered by these observers: *A. maculipennis*, *a. bifurcatus* and *A. nigripes.*

L. Legee found *a. maculipennis* in large numbers in the neighborhood of Grenoble and in the whole valley of the Isere ; this mosquito is very common in the low valley of the Drac, a region formerly malarious.

Hesse, quoted by Séger, has shown the presence of anopheles in great quantity in the districts of the peat-bogs of the Upper Saone from where malaria has disappeared for thirty years.

A. Sergent has found large numbers of anopheles (*A. maculipennis, A. bifurcatus*) along the banks of the Ersonne, in a region formerly malarious now healthy.

R. Blanchard has shown the presence of numerous anopheles

(*A. bifurcatus*), in a healthy region, at Charbouniéus, in the neighborhood of Lyons.

Br. Gallio Valerio has shown the presence of numerous anopheles (*A. maculipennis* and *A. bifurcatus*) in regions formerly infested with malaria (plains of the Rhone, the Orbe, valley of the Broye). The malarial fevers have disappeared to-day from these regions.

At Massarosa (Italy), anopheles are very numerous, according to Grassi; however, the malarial fevers are absent or exceedingly rare.

In certain localities of Italy, Celli and Gaspesini write, that all the conditions favorable to the development of malaria seem united: marshes, anopheles in large numbers, patients ill with malaria contracted at other places; and yet malarial fever is very rarely contracted there.

Schoo in Holland has found anopheles (*A. maculipennis*) in healthy as well as unhealthy localities.

Ed. and Etilenne Sergent found anopheles (*A. malculipennis* and *A. bifurcatus*) in the suburbs of Paris, along the woods of Boulogne, of Chalais, Villeneuve à Garches.

The existence of anopheles in the North of Europe in localities free from malaria is easily explained—the anopheles can not become infected by sucking the blood of patients ill with malarial fever; on the other hand the climatic conditions are little favorable to the development of *hemameba malariœ* in the bodies of the mosquitoes, for if by chance an anopheles did bite such malarial blood (from a patient entering the colonies, for example), there are many chances that the evolution of the parasite would not take place or would not get into the mosquito.

According to Koch a mean temperature of 25° C. would be necessary for the development of the *hemameba malariœ* in the anopheles.

The researches of Schoo in Holland prove that devolopment can occur at lower temperatures. At 18° C. the parasites develop well, the evolution is only slower than at 25° C.; an outside temperature which varies during the first days from 14° to 10° C. and later from 10° to 22°C. does not completely prevent the evolution of the parasite in the body of the mosquito.

It is understood that certain places are healthy in spite of the persistent presence of the anopheles; when, however, a population is poor and lives under bad hygienic conditions, in a swampy country, the sick are numerous, they are badly cared for and the anopheles become easily infected; if the general hygienic conditions are bettered, following the development of means of communication (roads and railroads), of progress shown in the cultivation of the soil, in the construction of good dwellings and in the food, the number of the sick decreases year by year; the physician is consulted when a case of fever occurs, quinine is widely distributed and the anopheles can no longer become infected. Drainage of the soil and drying up of the marshes have for their effect the diminution of the number of anopheles.

Such appears to be the explanation of the health of a large number of localities where the anopheles still are to be found.

All the anopheles do not appear to be equally able to propagate malaria, which accounts for the fact that certain localities in warm lands are healthy, although anopheles in great numbers are found.

Endemic malaria does not exist at Calcutta, however the anopheles are numerous in this city and the environs, but the anopheles is always the *A. Rossi* Giles, a species which seem to be but little dangerous from the standpoint of malarial propagation.

Stephens and Christophers have never found at Calcutta or in the environs *A. Rossi* naturally infected and they have not succeeded in infecting these mosquitoes by making them bite patients suffering from malarial fever.

The researches of James, made likewise in India, have confirmed the results of Stephens and Christophers. James never found *A. Rossi* infected naturally, even in very malarious localities where anopheles belonging to other species were often infected; he has succeeded, however, in infecting *A. Rossi* by making them bite patients ill with malaria. James supposes that in the free state these anopheles prefer the blood in animals to that of man.

All the observers who have studied the development of *H. malariæ* in the anopheles know that one frequently fails to infect these mosquitoes, even when favorable conditions of temperature are present. The anopheles either do not become infected or the infection miscarries; sporoblasts are found in the intestine, but the sporozoites do not pass to the salivary glands.

According to Schoo, anopheles fed on acid fruits do not become infected when made to bite patients ill with malaria; on the contrary, when they are given only water and for four days after sucking malarial blood, non-acid fruits, as, for example, melon, they become easily infected. The manner in which the anopheles are fed, then, exercises a marked influence on the evolution of the infection.

To sum up, the inquiry which has been pursued for several years in all parts of the world concerning the relations existing between mosquitoes and malaria has given results decidedly favorable to the doctrine of anophilism. Anopheles have been found in all malarial localities; every time that a statement brought forward contrary to this rule has been the object of a vigorous investigation, its inaccuracy has been proven. For endemic malaria to exist in a locality, the presence of anopheles alone is not sufficient; these mosquitoes must be capable of becoming themselves infected by sucking the blood of malarious patients. The fact, to-day well demonstrated, that the malarial parasite often exists in the blood of native children in a malarious country, is a fact of great interest.

The constant presence of anopheles in localities formerly unhealthy, to-day healthy, is oftenest explained by the hygienic progress in these localities; the inhabitants better housed, better nourished, better cared for when sick, making general use of quinine, are no longer a source of infection for the anopheles, which, therefore, cease to be dangerous. Sometimes this explanation does not take account of all the known facts; the harmlessness of the anopheles in certain localities, in certain conditions, has still other causes. All the anopheles are not equally capable ot propagating malaria; *A Rossi*, for example, appears harmless. There is still a field for continuing the researches already begun for the study of the conditions which favor the development of the *H. malariæ* in the body of the anopheles, or which place obstacles in the way of development.

Translator's note: The mosquitoes most concerned in propagating malaria in this country are *A. maculipennis* and *A. crucians*. *A. punctipennis* was also thought to be able to inoculate malaria, but a recent work by Heirschberg (Bul. Johns Hopkins Hospital, February, 1904) throws some doubt on this view. The *A. maculipennis* is without doubt the European name for our *A. quadrimaculatus*. In fact, recent writers in this country have altogether adopted the former name. This mosquito is widely spread over this country. It has been found abroad in summer, and in winter hibernating in barns and stables. *A. crucians* does not seem to be so wide-spread. Possibly more careful research will reveal new localities. It has been found in Georgia. I have found both kinds of anopheles around Savannah, together with large numbers of *A. punctipennis*.

